# Identity Leadership, Social Identity Continuity, and Well-Being at Work During COVID-19

**DOI:** 10.3389/fpsyg.2021.684475

**Published:** 2021-06-11

**Authors:** Henning Krug, S. Alexander Haslam, Kathleen Otto, Niklas K. Steffens

**Affiliations:** ^1^Institute of Psychology, Philipps University of Marburg, Marburg, Germany; ^2^School of Psychology, University of Queensland, St. Lucia, QLD, Australia

**Keywords:** COVID-19, social identity, identity leadership, identity continuity, health, well-being

## Abstract

The COVID-19 pandemic has led to widespread remote working that has posed significant challenges for people’s sense of connection to their workplace and their mental health and well-being. In the present work, we examined how leaders’ identity leadership is associated with the well-being of employees in the context of the COVID-19 pandemic. Specifically, we examined how both leaders’ and team members’ identity leadership is associated with employees’ social identity continuity, and through this with their job satisfaction, burnout and loneliness at work. Employees (*N* = 363) participated in a field study during the COVID-19 pandemic, completing measures of their leader’s and team members’ identity leadership (i.e., entrepreneurship and impresarioship), social identity continuity, job satisfaction, burnout, loneliness at work. Results revealed that to the extent that employees perceived greater social identity continuity, they were more satisfied with their work and felt less lonely. Furthermore, mediation analyses revealed indirect effects of team members’ identity entrepreneurship on job satisfaction and loneliness via an increase in social identity continuity. Results suggest that to foster employees’ health and well-being in times of disruption, organizations might put in place practices that allow employees to maintain a sense of ‘we-ness’ at work by involving not only formal leaders but also other members of the organization.

## Introduction

Since late January 2020, the world has been in the grip of the novel coronavirus (SARS-CoV-2) and the COVID-19 pandemic. According to the World Health Organization as of 23 March 2021, over 122 million people worldwide have been infected, while over 2.7 million people have died as a result of the disease (WHO, 2021). The lives of people around the world have been greatly impacted by the pandemic—not only by the risk of infection but also by changes to the way governments, communities and organizations operate. In particular, countries around the world have put in place a range of regulations to control the spread of the virus, including strict physical distancing measures and travel bans—also causing psychological distress (e.g., Bräscher et al., 2021). Moreover, the new regulations have brought about significant changes to people’s working lives: while health professionals have been working intensively on the frontline in hospitals, communities, and care homes, people in many other professions have been ordered to work from home in order to curb the spread of the virus.

In March 2020, 75.4% of German employees described themselves as working from home (Statista, 2020). Initial evidence suggests that by making it more difficult for people to connect and communicate with others in the workplace, these changes have posed a significant threat to people’s well-being (e.g., Qualtrics, 2020; Meyer et al., 2021; Möhring et al., 2021). However, we have little empirical knowledge of (a) the effects that changes in people’s working lives have had on their health and well-being and (b) the role that workplace leaders play in buffering any potential detrimental health effects.

In the present work, we examine these two key issues. First, how changes in working conditions have impacted on employees’ well-being. Second, whether any negative impacts can be counteracted by *identity leadership* behavior that focuses on creating and sustaining a sense of “us-ness” among workgroup members (Haslam et al., 2020). By this means we expand the growing literature informed by the social identity approach to leadership to examine the importance of identity leadership under remote working conditions. At the same time, this work explores points of contact between the social identity approach to leadership (which stresses the importance of cultivating a sense of “we” and “us” for effective leadership; Steffens et al., 2014; Haslam et al., 2020) and the “social cure” literature associated with the social identity approach to health (which highlights the importance of developing and maintaining groups memberships for health; Jetten et al., 2012; Haslam et al., 2018; Wakefield et al., 2019). More specifically, we seek to do this by examining the degree to which identity leadership supports team members’ well-being by contributing to their sense of social identity continuity under remote working conditions. On this basis, we also look to provide organizations and employees with practical advice informed by social psychological theorizing to help them adapt to challenges imposed by physical distancing and reduced opportunities for face-to-face contact in the workplace.

### Social Identity Continuity

The *social identity approach*, comprising *social identity theory* (Tajfel and Turner, 1979), and *self-categorization theory* (Turner et al., 1987), proposes that the self-concept of individuals not only rests on their *personal identity* (“I” or “me”) but also on their *social identity* (“we” or “us”) that is derived from memberships in social groups (Haslam, 2004). Research informed by the social identity approach indicates that when people see themselves as part of a group and identify with it (e.g., so that they see themselves as a member of a family, a work team or a community), they derive a range of important psychological resources from that group membership (Haslam et al., 2018). This is because it is through social identity that people experience psychological connection to fellow group members (Haslam et al., 2021), that provide for social support (Levine et al., 2002; Haslam et al., 2005), a sense of meaning and purpose (van Dick and Wagner, 2002; Wegge et al., 2006), and a sense of control (Greenaway et al., 2015; see also Greenaway et al., 2016). In light of these benefits, people’s sense of belongingness to, and identification with, social groups has been shown to have important benefits for health and well-being more generally (Tewari et al., 2012; Cruwys et al., 2014; Haslam et al., 2018; Postmes et al., 2019; Wakefield et al., 2019). This is true for groups in society at large but also for groups in the workplace (e.g., work unit, teams, departments, or whole organizations; Avanzi et al., 2015; Karanika-Murray et al., 2015; Steffens et al., 2017).

Moreover, research suggests that identifying with *multiple* social groups further increases well-being because this generally provides people access to more social identity-based psychological resources. It also means that if one group membership is lost (e.g., as a result of organizational change or a life transition such as retirement; Steffens et al., 2016; Haslam C. et al., 2019), a person will have other groups to fall back on and buffer them against the psychological fallout from that change (Jetten et al., 2015; Haslam et al., 2021).

There are reasons for supposing, however, that COVID-19 threatens people’s access to these group-related resources (see Jetten et al., 2020; van Bavel et al., 2020, for reviews). In particular, this is because during lockdown many people have been required to work from home and thus have not been able to come together with their various work-related groups as they did to prior to the pandemic. Others are still able to go to their physical place of work but are nevertheless affected by new regulations which require them to engage in social distancing. As a result of these regulations, many employees will also not be able to partake in activities that had been inherent to their membership of a particular workplace group (e.g., team meetings, conferences, social gatherings), while for others face-to-face interactions have shifted completely “online.”

Overall, the changes wrought by COVID-19 would be expected to impact negatively on a sense of *social identity continuity* associated with ongoing membership of a particular organizational unit (Sani et al., 2008; Haslam et al., 2021). In particular, it seems likely that the changes to working practice brought about by the pandemic will have disrupted the range of activities, rituals, and practices which help to keep teams functioning *as teams* (Haslam, 2004). Research on the social identity approach to health thus leads us to expect that this will tend to compromise health and well-being. More formally, we hypothesize:

*Hypothesis 1:* The more social identity continuity employees experience in their work-related group memberships, the better their work-related well-being will be in terms of (a) higher job satisfaction, (b) lower loneliness at work, and (c) lower burnout.

### Identity Entrepreneurship and Identity Impresarioship

Yet while research on social identity and health leads us to expect that the pandemic compromised health and well-being by undermining people’s ability to maintain valued group memberships at work, work on social identity and leadership also points to the role that leaders and other group members can play in promoting health in the workplace (Steffens et al., 2014; van Dick et al., 2018; Haslam et al., 2020). This, then, provides insights into ways that groups may be able to offset the potentially negative health effects of the current crisis. More specifically, research suggests that leaders (both formal and informal; D’Innocenzo et al., 2016) can do this by engaging in *identity leadership* that helps to (re)build a sense of social identity in the workplace (e.g., with a team, unit, or the organization as a whole; Steffens et al., 2014; Haslam et al., 2020).

In the context of disruptions caused by COVID-19, two dimensions of identity leadership that seem especially likely to be important are leaders’ identity entrepreneurship and identity impresarioship. This is because these dimensions comprise behaviors that are likely to help employees maintain the sense of what ‘us’ actually means while enabling them to come together and enact their group membership as they experience disruption. More specifically, *identity entrepreneurship* refers to behaviors that aim to increase group cohesion as well as group members’ understanding of what a group is about and what it stands for (e.g., its norms and values) in ways that help to “craft a sense of us” (Reicher and Hopkins, 2001; Reicher et al., 2005; Steffens et al., 2014). Previous longitudinal research has shown that such behavior has the capacity not only to increase group members’ engagement in group activities but also to improve their health and well-being—notably by reducing burnout (e.g., Steffens et al., 2018; Fransen et al., 2020). However, identity entrepreneurship would seem to be important in the context of the disruption to working life brought about by COVID-19 since, as noted above, it seems likely that changes to working arrangements have compromised workplace social identity by compromising established patterns of group communication and connection. On this basis, we hypothesize that:

*Hypothesis 2a:* Identity entrepreneurship on the part of the leader will be positively associated with (a) higher job satisfaction, (b) lower burnout, and (c) lower loneliness at work through an increased sense of social identity continuity in work-related group memberships.
*Hypothesis 2b:* Identity entrepreneurship on the part of other team members will be positively associated with (a) higher job satisfaction, (b) lower burnout, and (c) lower loneliness at work through an increased sense of social identity continuity in work-related group memberships.

Yet in addition to leaders’ efforts to create or maintain a sense of “us” in the way that they engage with groups, their efforts to put in place structures, events, and activities that *embed* a sense of shared identity should be important in times of the current pandemic, too. For these acts of *identity impresarioship* allow the “idea of us” to be translated in material reality (Haslam et al., 2020). During COVID-19, this might comprise a range of initiatives that create opportunities and environments for employees to come together as a group and live out their shared group membership (e.g., through regular meetings and events, even if these are only virtual). Again, there is evidence in other contexts that this is important both for engagement and for health and well-being, again because it helps group members to (re)gain a sense of identity continuity (Stevens et al., 2018, 2020; van Dick et al., 2018). On this basis, we hypothesize that:

*Hypothesis 3a:* Identity impresarioship on the part of the leader will be positively associated with (a) higher job satisfaction, (b) lower burnout, and (c) lower loneliness at work through an increased sense of social identity continuity in work-related group memberships.
*Hypothesis 3b:* Identity impresarioship on the part of other team members will be positively associated with (a) higher job satisfaction, (b) lower burnout, and (c) lower loneliness at work through an increased sense of social identity continuity in work-related group memberships.

## Materials and Methods

### Sample and Data Collection

Data was collected between April 11th and May 2nd 2020. Participants were recruited using snowball sampling via social media and mailing lists. The local ethics committee of the first author’s university granted ethical approval and informed consent was obtained from all participants. Three-hundred-and-sixty-seven employees completed the questionnaire, all of whom reported being currently employed and as having colleagues as well as a formal leader at work. Data from four participants were excluded due to a completion time that suggested careless responding (<5 min total). Thus, the final sample consisted of *N* = 363 employees. Participants’ age ranged from 19 to 63 (*M* = 36.31, *SD* = 11.01) and 68% were female. Participants’ average organizational tenure was 7.90 years (*SD* = 8.20) and 62.8% worked full-time. Most frequently indicated industries that participants worked in were education and science (16.8%), public service (15.4%), health and social work (15.2%), manufacturing and engineering (11%), and automotive industry (6.6%). To determine whether participants were confident with remote working conditions, we asked them about the frequencies with which they used different channels of communications in their work. We also asked how that had changed through COVID-19. On average, 74.24% of the total communication pre-COVID-19 was digital, while 87% of the total communication was digital during COVID-19. Thus, participants were already largely familiar with digital communication pre-COVID-19 but the share of digital communication had increased during COVID-19. More information on the direct impact of COVID-19 experienced by the participants is provided in Table 1.

**TABLE 1:** Work-related changes due to the COVID-19 pandemic experienced by participants.

**Changes due to the COVID-19 pandemic**	**Number of participants (percentage)**
**Working hours**	
More	62 (17.1%)
Less	116 (32.0%)
Same	185 (51.0%)
**Home office**	
Not at all	101 (27.8%)
A little	47 (12.9%)
Mostly	64 (17.6%)
Completely	151 (41.6%)
Short-time work	48 (13.2%)
Childcare at home	67 (18.5%)

### Measures

#### Identity Leadership by Formal Leaders and Fellow Team Members

Identity entrepreneurship and impresarioship were measured with four items using adapted items from the German version of the Identity Leadership Inventory (Steffens et al., 2014; van Dick et al., 2018). Responses were made on a scale from 1 (*totally disagree*) to 7 (*totally agree*). Participants were asked to rate both their leader and their fellow team members on these items. A sample item for identity entrepreneurship was “This leader makes/members of my team make people feel as if they are part of the same group in times of the coronavirus.” Cronbach’s α for this subscale was 0.96 for the leader-related items and 0.95 for the team-related items. A sample item for identity impresarioship was “This leader devises/members of my team devise virtual activities that bring the team together in times of the coronavirus.” Cronbach’s α for this subscale was 0.89 for the leader-related items and 0.94 for the team-related items. Compared to the original items for impresarioship (Steffens et al., 2014; van Dick et al., 2018), our items referred to *virtual* activities/events/structures created by the leader to account for the changed, more remote work environment during the pandemic. An English translation of the adapted impresarioship items can be found in Appendix A.

#### Perceived Social Identity Continuity at Work

This was measured using a German adaptation of the four-item social identity continuity measure from Haslam et al.’s (2008) Exeter Identity Transition Scales (EXITS) Responses were made on a scale from 1 (*totally disagree*) to 7 (*totally agree*). A sample item was “Since the outbreak of the coronavirus, I still belong to the same groups at work I was a member of before the outbreak” (α = 0.76).

#### Job Satisfaction

This was assessed with a single five-grade Kunin-item (Baillod and Semmer, 1994; “All in all, how satisfied are you with your job?”).

#### Burnout

This was measured with six items from the German version of the Copenhagen Burnout Inventory (CBI; Kristensen et al., 2005; Nübling et al., 2005; sample item: “How often do you feel tired?”). Responses were made on a scale from 1 (*almost never/never*) to 5 (*always*; α = 0.87).

#### Loneliness at Work

To measure loneliness at work, a German adaptation of four items from Ozcelik and Barsade’s (2018) Workplace Loneliness Scale (sample item: “In this organization, I can find companionship when I want it”). Responses were made on a scale from 1 (*totally disagree*) to 7 (*totally agree*; α = 0.78).

## Results

Means, standard deviations, and intercorrelations are presented in Table 2. Hypotheses were assessed with structural equation modeling (SEM) using Amos (Arbuckle, 2014).

**TABLE 2:** Descriptive statistics and correlation coefficients.

**Variable**	***M***	***SD***	**1**	**2**	**3**	**4**	**5**	**6**	**7**
1. Identity entrepreneurship–leader	4.69	1.61	–						
2. Identity impresarioship–leader	3.85	1.78	0.57**	–					
3. Identity entrepreneurship–team	4.98	1.31	0.48**	0.35**	–				
4. Identity impresarioship–team	4.12	1.74	0.31**	0.49**	0.57**	–			
5. Social identity continuity	5.20	1.16	0.30**	0.27**	0.38**	0.28**	–		
6. Job satisfaction	3.82	0.81	0.43**	0.24**	0.28**	0.18**	0.26**	–	
7. Burnout	2.57	0.71	−0.15**	−0.06	−0.06	−0.04	−0.08	−0.34**	–
8. Loneliness at work	2.85	1.09	−0.29**	−0.18**	−0.51**	−0.32**	−0.29**	−0.33**	0.12*

N = 361–363. **p < 0.01 (two-tailed). *p < 0.05 (two-tailed).

Before testing our hypotheses, we ran a confirmatory factor analysis (CFA) to evaluate the measurement model (cf. Huang et al., 2017). The CFA of a 7-factor model that included latent factors of identity entrepreneurship (leader/team), identity impresarioship (leader/team), social identity continuity, loneliness, and burnout indicated that the model had a sufficient fit to the data, *x^2^* = 965.459, *df* = 357, TLI = 0.91, CFI = 0.92, RMSEA = 0.07. Furthermore, in order to see whether our 4-factor solution regarding identity leadership (identity entrepreneurship leader, identity entrepreneurship team, identity impresarioship leader, identity impresarioship team) is adequate, we conducted a CFA to compare the 4-factor model with a 2-factor model (identity leadership by leader, identity leadership by team members; *x^2^* = 1513.932, *df* = 76, TLI = 0.681, CFI = 0.734, RMSEA = 0.229) and with a 4-factor model with a second order factor (*x^2^* = 368.866, *df* = 72, TLI = 0.930, CFI = 0.945, RMSEA = 0.107). The proposed 4-factor-model had a significantly better fit (*p* < 0.001) than the other two models, which is why we continued with this model for the analysis. Moreover, we employed Harman’s one-factor test to check if common method variance is likely to bias the results (Podsakoff and Organ, 1986). Since there was no factor that accounted for more than 50% of the variance (six factors with an eigenvalue > 1 were extracted; the first factor accounted for 30.66% of the variance), it can be assumed that common method variance is unlikely to be a problem (cf. Fuller et al., 2016).

### Structural Models

**FIGURE 1:**
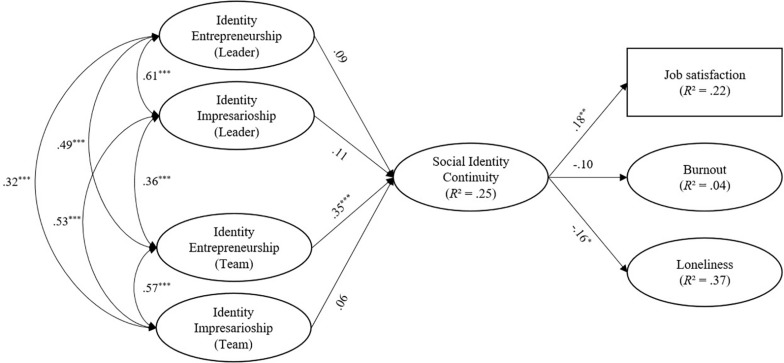
Standardized effects and correlations from the structural equation model. *N* = 361. *p < 0.05, **p < 0.01, ***p < 0.001. Direct effects of IVs are not displayed for the sake of clarity. See Table 3 for direct effects.

We compared the fit of our hypothesized structural equation model against two alternative models: One was a *“no directs”* model with *no direct effects* of identity leadership on health and well-being variables (*df* = 365; *x^2^* = 1000.372; TLI = 0.91; CFI = 0.92; RMSEA = 0.07) the other was an “*only directs”* model with *only direct effects* of identity leadership on health and well-being variables (*df* = 360; *x^2^* = 973.438; TLI = 0.91; CFI = 0.92; RMSEA = 0.07), where the mediator (social identity continuity) was included in the model with no paths stemming from or leading to it (cf. Mathieu and Taylor, 2006). The alternative models yielded a worse fit (*p* < 0.001) than the baseline model (*df* = 353; *x^2^* = 885.955; TLI = 0.92; CFI = 0.93; RMSEA = 0.07). Accordingly, we proceeded to test the hypotheses specified in our proposed structural equation model. The standardized effects of the proposed indirect effects are displayed in Figure 1 while the standardized effects of the direct effects from the antecedents to the outcome variables are reported in Table 3. The bootstrapped 95% confidence intervals for the hypothesized indirect effects are reported in Table 4.

**TABLE 3:** Standardized direct effects of identity entrepreneurship and impresarioship (leader/team) on outcomes from structural equation model.

	***ß***	***p***
**Job satisfaction**		
Identity entrepreneurship leader	0.41 (0.07)	<0.001
Identity impresarioship leader	−0.08(0.08)	0.286
Identity entrepreneurship team	0.02 (0.08)	0.819
Identity impresarioship team	0.03 (0.08)	0.656
**Burnout**		
Identity entrepreneurship leader	−0.23(0.09)	0.006
Identity impresarioship leader	0.14 (0.10)	0.133
Identity entrepreneurship team	0.06 (0.09)	0.444
Identity impresarioship team	−0.03(0.09)	0.682
**Loneliness at work**		
Identity entrepreneurship leader	−0.17(0.09)	0.025
Identity impresarioship leader	0.17 (0.09)	0.042
Identity entrepreneurship team	−0.42(0.08)	<0.001
Identity impresarioship team	−0.11(0.08)	0.162

*N = 361. Standardized regression weights reported with standard errors in parentheses.*

**TABLE 4:** Standardized indirect effects of identity entrepreneurship and impresarioship (leader/team) on outcomes at work via social identity continuity.

	**BCaCI**
**Outcome: job satisfaction**	
IL-E leader → SIC → JS	[−0.01, 0.07]
IL-I leader → SIC → JS	[−0.01, 0.07]
IL-E team → SIC → JS	[0.01, 0.14]
IL-I team → SIC → JS	[−0.01, 0.05]
**Outcome: burnout**	
IL-E leader → SIC → BO	[−0.06, 0.01]
IL-I leader → SIC → BO	[−0.06, 0.01]
IL-E team → SIC → BO	[−0.12, 0.02]
IL-I team → SIC → BO	[−0.05, 0.01]
**Outcome: loneliness at work**	
IL-E leader → SIC → LO	[−0.07, 0.01]
IL-I leader → SIC → LO	[−0.07, 0.00]
IL-E team →SIC → LO	[−0.15, -0.01]
IL-I team → SIC → LO	[−0.06, 0.01]

N = 361. BCaCI = 95% bias corrected and accelerated confidence intervals. Confidence intervals based on 10,000 bootstrap samples. IL-E Leader, identity entrepreneurship shown by leader; IL-E Team, identity entrepreneurship shown by team; IL-I Leader, identity impresarioship shown by leader; IL-I Team, identity impresarioship shown by team; SIC, social identity continuity; JS, job satisfaction; BO, burnout; LO, loneliness at work.

#### Tests of H1

We hypothesized that employees who experienced more social identity continuity in their work-related group memberships during COVID-19, would report higher work-related well-being. As shown in Figure 1, we found partial support for this hypothesis. More specifically, social identity continuity was significantly associated with employees’ (a) greater job satisfaction and (c) lower loneliness but not with (b) reduced burnout.

#### Tests of H2

We hypothesized that identity entrepreneurship on the part of the leader (H2a) and other team members (H2b) would be associated with (a) higher job satisfaction, (b) lower burnout, and (c) lower loneliness at work, mediated by employees’ experience of social identity continuity in their work-related group memberships. Results revealed two significant indirect effects as displayed in Table 4. In partial support of H2b, identity entrepreneurship on the part of by team members was associated with a stronger sense of social identity continuity among respondents and, through this, with (a) greater job satisfaction and (c) lower loneliness. H2a was therefore not supported.

#### Tests of H3

We hypothesized that identity impresarioship on the part of the leader (H3a) and other team members (H3b) would be associated with (a) higher job satisfaction, (b) lower burnout, and (c) lower loneliness at work mediated by employees’ experience of social identity continuity in their work-related group memberships. There was no evidence that identity impresarioship (on the part of either team members or leaders) was associated with a stronger sense of social identity continuity among respondents. Accordingly, there was no support for H3a and H3b.

#### Controlling for Organizational Tenure

As suggested by a reviewer, we added organizational tenure as a control variable to the model to examine whether employees’ socialization stage within the organization affects the relationships. After including organizational tenure as a control variable in the model, the overall results pattern regarding the hypothesized relationships did not change. Only the indirect effects of identity entrepreneurship by team members via social identity continuity on job satisfaction and loneliness were now marginally significant (*p* = 0.052 for the indirect effect on loneliness; *p* = 0.065 for the indirect effect on job satisfaction). However, this small change in the significance levels might be due to a lower power because the sample size decreased to *N* = 315 due to missing values on the organizational tenure variable. We further inspected bivariate correlations and organizational tenure was not significantly related to any of the variables included in the model. Thus, it can be concluded that organizational tenure did not substantially influence the results.

## Discussion

In the present research, we drew on the social identity perspective to explore predictors of the work-related well-being of employees in the context of the COVID-19 pandemic. Specifically, this study was designed to examine the role of people’s sense of social identity continuity in work-related group membership in supporting well-being during the pandemic. In support of H1a and H1c, results indicated that work-related social identity continuity was related to increased job satisfaction and reduced loneliness among employees (while there was no relationship with burnout; H1b). Moreover, we examined whether identity entrepreneurship (e.g., Reicher and Hopkins, 2001; Reicher et al., 2005; Haslam et al., 2020) and identity impresarioship (Steffens et al., 2014; Haslam et al., 2020) as shown by the formal leader and other team members might play a role in fostering this sense of identity continuity. Results provided evidence of an indirect effect of identity entrepreneurship shown by team members (but not by the leader) on job satisfaction and loneliness (thereby providing partial support of H2a and H2c). All other indirect effects were non-significant and, in particular, they provided no support for H3 (i.e., of identity impresarioship as a predictor of identity continuity, and, through this, of job satisfaction, burnout, and loneliness at work).

### Theoretical and Practical Implications

The present research has several implications for theory as well as practice. First, our results underline the importance of people’s sense of social identity continuity (e.g., Sani et al., 2008; Herrera et al., 2011) for employees’ well-being in times of crisis and disruption. In this regard, our research also expands upon previous research that has highlighted the importance of social identity continuity for well-being during major life changes such as transitioning to university life (Iyer et al., 2009), recovering from collective trauma (Muldoon et al., 2017), becoming a mother (Seymour-Smith et al., 2017), retiring from work (Steffens et al., 2016), moving overseas (Cruwys et al., 2020), and recovering from illness (Haslam et al., 2008; for a review, see Haslam et al., 2021). Like many of these transitions, the disruptive changes brought about by COVID-19 have meant this has been a life-changing “once-in-a-lifetime” event and in this context, too, it appears that the maintenance of workgroup memberships has had a significant role to play in reducing people’s loneliness and helping to sustain their life satisfaction.

The results of our research have important implications for leaders in organizations as well. In particular, by pointing to the importance of social identity continuity for well-being at work in times of change, it provides organizations with a guiding framework for understanding how to maintain their employees’ health and engagement during the current (and possibly other) crises. Here or results show that if employees are able to stay connected with their work-related groups, this can support their well-being in the face of the range of challenges brought about by this crisis.

In this context, just as our research contributes to work on social identity and health, so too it advances a growing body of work on social identity approach to leadership (Steffens et al., 2014; van Dick et al., 2018; Haslam et al., 2020). While previous research has demonstrated the positive impact of leaders’ engagement in identity leadership on group members’ engagement and burnout (Steffens et al., 2018) and health (Fransen et al., 2020), the present research indicates that identity leadership can also help to minimize employees’ feeling of alienation and loneliness at work (see also Seppala and King, 2017). Here our findings also align with emerging evidence that identity leadership that is *shared* has unique benefits for teams. For instance, Fransen et al. (2020) found that sport team members’ health was impacted as much by the leadership of the formal leader as it was by the leadership of informal leaders. Extending this body of work, the present work found that it was perceptions of identity entrepreneurship on the part of fellow team members that was associated with members’ perceived continuity of work groups and greater satisfaction with work and lower loneliness. Accordingly, it seems that in times of crisis such as the present pandemic, well-functioning teams will be those in which *all* team members help to create a sense of togetherness that contributes to employees’ sense of identity continuity and well-being at work. Looking at the direct effects of identity entrepreneurship on employees’ well-being (cf. Table 3), we see that formal leaders’ entrepreneurship is associated with higher well-being of employees. However, these relationships are likely to involve a different mechanism (i.e., other than social identity continuity). These findings suggest that organizations should not rely only on formal leaders to maintain a shared sense of “us” in times of crisis, but should also encourage other members of the organization to do the same. Indeed, the strong association between the identity leadership of leaders and that of team members suggests that this may be an important (and hitherto unstudied) aspect of identity leadership.

In the case of identity impresarioship, however, we did not find any significant support for our hypotheses. It is noteworthy, too, that while impresarioship was associated with greater job satisfaction and lower loneliness (see Table 2), these correlations were weaker than those for entrepreneurship, suggesting that entrepreneurship may be more important than impresarioship for employees’ health and well-being. Moreover, the modeling results revealed a significant, *positive* relationship between leaders’ identity impresarioship and increased loneliness of employees (cf. Table 3). This suggests that there may be circumstances under which leaders’ efforts to create structures, activities, and events may *add to* employees’ stress and alienation during a crisis—possibly because these are a source of (additional) demand rather than a resource (which is seen to control rather than support). These findings do not necessarily suggest that structures and activities around the group are bad for the health of team members, but they do suggest that leaders may not always have a good sense of what sorts of activities support team functioning and health under the present conditions. Going forward, it is going to be important to provide greater insight into ways that these material actions can be structured so as to lock in the benefits of the shared identity rather than to scuttle them.

### Limitations and Future Research

Of course, our research is not without limitations. Most obviously, our results are based on cross-sectional field data, which makes causal inferences impossible. However, given the novelty of the current circumstances and the novelty of the theoretical model that we proposed and examined, it seems justified to seek to provide initial insight into the issues we were addressing by means of cross-sectional investigation. Nevertheless, future research should employ longitudinal and intervention designs to further assess the impact of the present relationships in related contexts—especially since many of the changes brought about by COVID-19 (e.g., an increase of digital vs. face-to-face communication and home office arrangements) seem likely to endure beyond the pandemic.

The measure of identity impresarioship that we used in the present research may also have been suboptimal. Here we adapted previously validated items from the Identity Leadership Inventory (Steffens et al., 2014; van Dick et al., 2018) in order to fit the COVID-19 context, with a view to capturing identity impresarioship in a virtual environment. However, this adaptation may have meant that we measured a somewhat different construct here. Suggestive of this, the correlations between the impresarioship and entrepreneurship were markedly lower than in previous studies (Steffens et al., 2014; van Dick et al., 2018). Future research is needed to clarify this issue, and might also compare how different aspects of identity leadership play out in different contexts (e.g., face-to-face vs. virtual) to establish when they are more likely to have beneficial consequences for health.

Another issue with regard to the measurement of leadership variables in the current study is that of multiple team membership (cf. O’Leary and Woolley, 2011) where employees have different potential supervisors that impact their work. Future research might therefore explicitly assess multiple team membership of participants to examine how employees are impacted by leadership of multiple teams.

## Conclusion

The present research sheds light on ways in which COVID-19 has impacted on employees’ health and well-being by compromising their sense of social identity continuity in the workplace. It also speaks to the capacity for identity leadership—especially on the part of other team members—to buffer employees from the impact of identity discontinuity by cultivating a sense of shared social identity in the workplace. In this, the findings speak to claims that social identity is critical not only for leadership but also for health. Indeed, precisely because identity leadership centers around the creation of shared sense of “we” and “us,” it can also be an important way of staving off the health-threatening effects of social disconnection and isolation (e.g., in the form of loneliness; Holt-Lunstad et al., 2010; Haslam S. A. et al., 2019). In the context of a pandemic, whose effects have been felt as much through increased physical distancing as through the spread of the virus itself, this capacity to build social connection and solidarity would seem to be particularly important. Moreover, because the need to build and maintain social identity is so great (Jetten et al., 2020), it is important to recognize that this is a task that should not be left to leaders to perform on their own. After all, if it is the case that “we are all in this together”, we all need to be in the business of making this call to solidarity ring true.

## Data Availability Statement

The datasets for this manuscript are not publicly available. Requests to access the datasets should be directed to HK, henning.krug@uni-marburg.de.

## Ethics Statement

The studies involving human participants were reviewed and approved by the Local Ethics Committee of the Institute of Psychology, University of Marburg, Germany. The participants provided their written informed consent to participate in this study.

## Author Contributions

HK, SAH, KO, and NS developed the study concept, designed the research, and edited the manuscript. HK performed the statistical analyses and drafted the manuscript. All authors read and approved the final manuscript.

## Conflict of Interest

The authors declare that the research was conducted in the absence of any commercial or financial relationships that could be construed as a potential conflict of interest.

## Appendix A

### Adapted Items for Identity Impresarioship (Steffens et al., 2014; van Dick et al., 2018)

This leader…

… devises virtual activities that bring the team together in times of the coronavirus.
… arranges virtual events that help the team function effectively in times of the coronavirus.
… creates virtual structures that are useful for the group in times of the coronavirus.

Members of my team…

… devise virtual activities that bring the team together in times of the coronavirus.
… arrange virtual events that help the team function effectively in times of the coronavirus.
… create virtual structures that are useful for the team members in times of the coronavirus.
